# The Role of Double Excitations in Exciton Dynamics of Multiazobenzenes: Trisazobenzenophane as a Test Case

**DOI:** 10.1021/acs.jpclett.4c01608

**Published:** 2024-07-16

**Authors:** Evgenii Titov

**Affiliations:** University of Potsdam, Institute of Chemistry, Theoretical Chemistry, Karl-Liebknecht-Straße 24-25, 14476 Potsdam, Germany

## Abstract

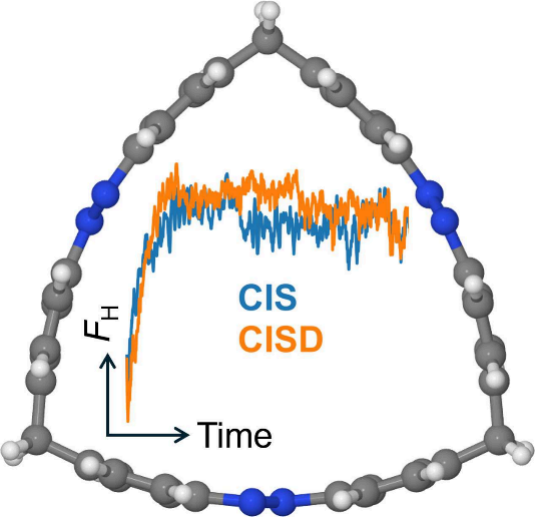

Molecular exciton dynamics underlie energy and charge transfer processes in organic multichromophoric systems. A particularly interesting class of the latter is multiphotochromic systems made of molecules capable of photochemical transformations. Exciton dynamics in assemblies of photoswitches have been recently investigated using either the molecular exciton model or supermolecular configuration interaction (CI) singles, both approaches being based on a semiempirical Hamiltonian and combined with surface hopping molecular dynamics. Here, we study how inclusion of double excitations in nonadiabatic dynamics simulations affects exciton dynamics of multiazobenzenes, using trisazobenzenophane as an example. We find that both CI singles and CI singles and doubles yield virtually the same time scale of dynamical exciton localization, ∼50 fs for the studied multiazobenzene. However, inclusion of double excitations considerably affects the excited state lifetimes and isomerization quantum yields.

Multichromophoric systems enable energy and charge transfer processes, which are ubiquitous in biological complexes and organic materials. On a fundamental level, assembling several (or many) chromophoric units together results in formation of molecular exciton states—the electronically excited states of the multichromophoric system.^1,2^ These states may be either delocalized over or localized on individual chromophores depending on the nature of individual constituents (homooligomer vs heterooligomer)^3^ and interplay between structural distortion and electronic coupling.^4,5^ Moreover, upon excitation with light, the exciton states may undergo ultrafast “dynamical” localization during excited state dynamics.^6−8^ A special class of multichromophoric systems is multi*photochromic* systems featuring photoswitchable molecular units combined together in a single compound bearing a potential to expand switching functionality.^9^ For example, various multiazobenzenes tailored to applications ranging from energy storage to wavelength-selective control of molecular switching have been devised to date.^10−16^ Also, a few theoretical, nonadiabatic dynamics studies have been performed with focus on photoreaction mechanisms, quantum yields, and excited state lifetimes.^17−20^ However, the exciton dynamics in systems with several azobenzene chromophores have been paid due attention only recently.^21−24^

Specifically, Sangiogo Gil, Persico, and Granucci used surface hopping combined with an *exciton model* to simulate Frenkel exciton dynamics in bisazobenzenophane^21^ and an azobiphenyl monolayer.^22^ Moreover, we studied exciton localization and exciton dynamics in (noncovalent) H-type azobenzene tetramers using a *supermolecule* surface hopping approach at the semiempirical configuration interaction singles (CIS) level.^23,24^ In particular, our simulations revealed ultrafast, sub-100 fs dynamical exciton localization after ππ* excitation of the tetramers.^23^ While CIS is a computationally and conceptually attractive approach, it misses doubly (and higher) excited states, e.g., the singlet correlated triplet pair,^25,26^ which may play a role in nonadiabatic relaxation. Thus, a question arises as to how exciton dynamics and, in particular, the localization time scale are affected by inclusion of higher excitations.

Here, we study the effect of double excitations on the exciton dynamics of a trisazobenzenophane^11,27^—a molecular ring composed of three azobenzene units, which are connected by CH_2_ groups in our case; see Figure 1, left.

**Figure 1:**
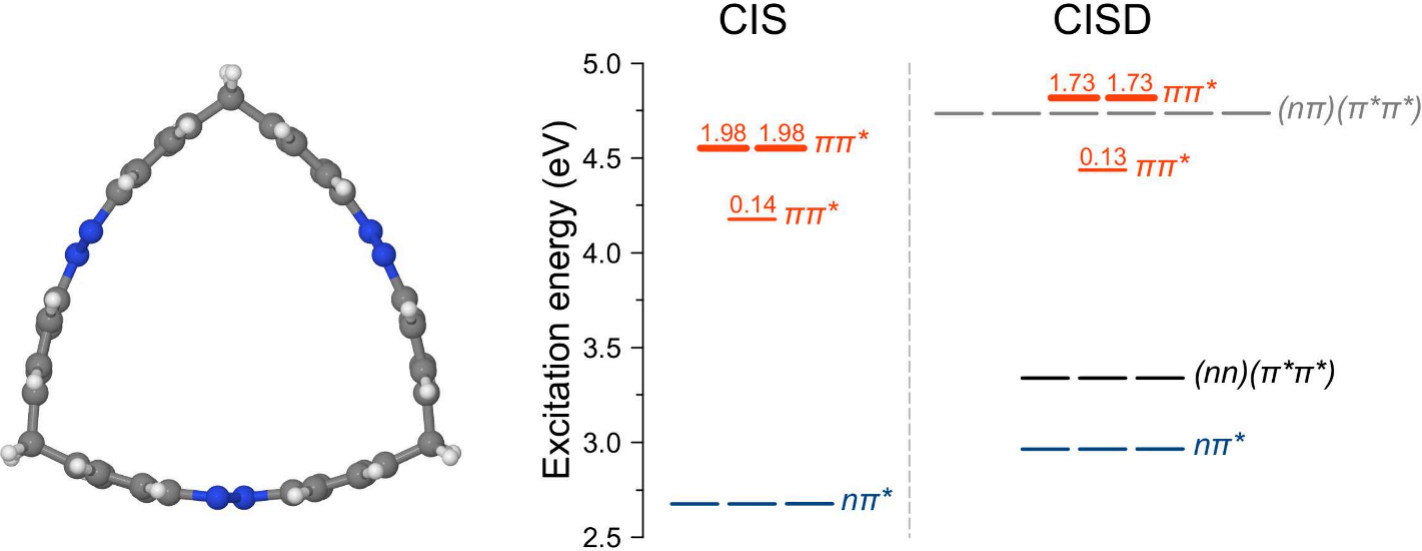
Left: Molecular structure of the studied trisazobenzenophane. Right: Excited state diagrams computed with the semiempirical CIS and CISD methods. The state characters are indicated. The doubly excited states are labeled as (*oo*′)(*vv*′), where electrons are excited from orbitals *o* and *o*′ to orbitals *v* and *v*′. The red numbers are the oscillator strengths for the ππ* transitions (other states are dark). The CIS calculation is performed at the CIS ground state optimized geometry; the CISD calculation at the CISD ground state optimized geometry.

Excited state diagrams obtained at the semiempirical rAM1-FOMO/CIS and rAM1-FOMO/CISD levels (referred to as “CIS” and “CISD”, respectively, in what follows) are shown in Figure 1, right [see the Methods section for details]. In both calculations, we use a restricted active space composed of three π orbitals (HOMO–5, HOMO–4, HOMO–3), three *n* orbitals (HOMO–2, HOMO–1, HOMO), and three π* orbitals (LUMO, LUMO+1, and LUMO+2); see Figure S1. [Here, HOMO is the highest occupied molecular orbital and LUMO is the lowest unoccupied molecular orbital.]

At the CIS level, we observe a picture which can be expected from the exciton model:^28^ Three low-lying states originating from the monomeric nπ* state and three higher lying states corresponding to the monomeric ππ* state. The nπ* states of the ring are virtually degenerate owing to small exciton coupling, whereas an exciton splitting of ∼0.37 eV is observed between the lowest ππ* state and the two (degenerate) upper ones.

At the CISD level, apart from the singly excited nπ* and ππ* states, which are somewhat blue-shifted in comparison to the CIS result, doubly excited states appear in the spectrum. Specifically, we find three (*nn*)(π*π*) states slightly above the nπ* states and six (*nπ*)(π*π*) states just below the brightest ππ* states.

A question arises of how these states affect the nonadiabatic dynamics of the multiazobenzene and, in particular, the dynamics of exciton localization. To tackle this question, we performed surface hopping (SH) simulations with both the CIS and CISD methods. In the case of CIS, six excited states and the ground state were included in the simulations. And in the case of CISD, 15 excited states and the ground state were accounted for (compare to Figure 1). The initial conditions (geometries and velocities) for the SH simulations were sampled from 20 ps long Langevin trajectories (at *T* = 300 K). The SH trajectories (100 for each system) were launched from the brightest ππ* state (see absorption spectra in Figures S2–S4 and initial populations in Table S1) and propagated for 10 ps. We note that the ordering of the excited states is very sensitive to the geometry. As a result, states *S*_8_–*S*_15_ are initially populated at the CISD level (Table S1). For comparison, we simulated nonadiabatic dynamics of the monomer using either CIS or CISD and the active space of three orbitals (π, *n*, and π* corresponding, respectively, to HOMO–1, HOMO, and LUMO of the monomer; see Figure S1). See the Methods section for further details.

The electronic state populations are shown in Figure 2.

**Figure 2:**
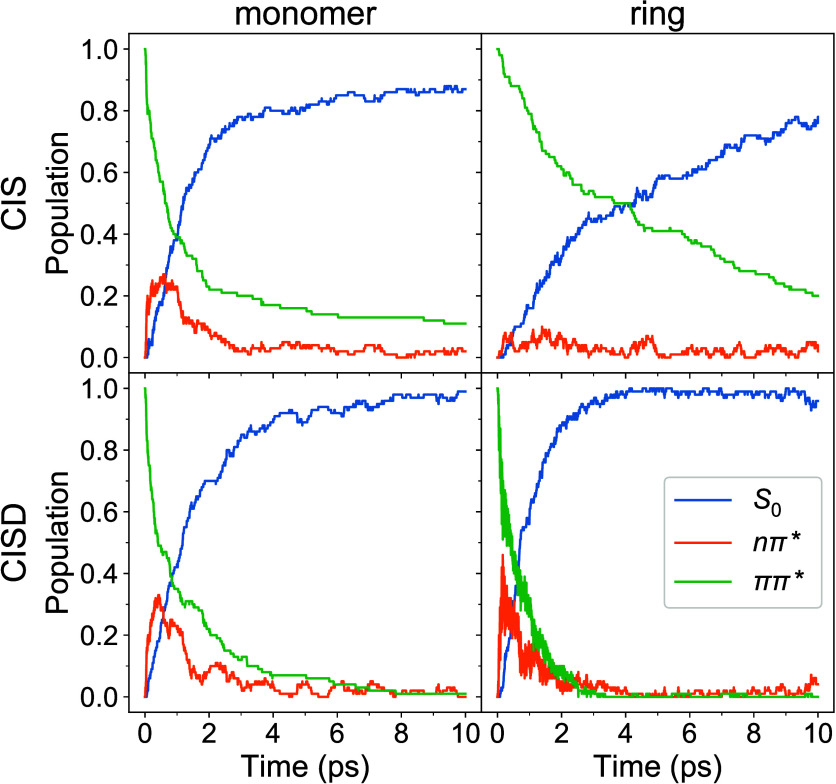
Electronic state populations for the monomer (left column) and the ring (right column) computed at the CIS (top row) and CISD (bottom row) levels of theory.

For the monomer, the *S*_1_ state is the nπ* state and the *S*_2_ state is the ππ* state. In the case of trisazobenzenophane, we group the excited states as follows. For CIS, *S*_1_–*S*_3_ build the nπ* manifold and *S*_4_–*S*_6_ the ππ* manifold. For CISD, *S*_1_–*S*_6_ are assigned to the nπ* manifold and *S*_7_–*S*_15_ to the ππ* manifold. We note that the last group also includes the (*nπ*)(π*π*) doubly excited states, but we term this group a “ππ*” manifold for simplicity (note that the (*nπ*)(π*π*) states are located near the ππ* states; see Figure 1). Detailed analysis of contributions of single and double excitations is provided in section S5 of the SI. This analysis shows the dominance of the single excitations throughout the dynamics (see Figures S9 and S10). The *S*_0_, nπ*, and ππ* populations were fitted using a two-step irreversible kinetic model ππ* → nπ* → *S*_0_:
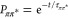
1a
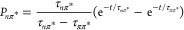
1b
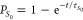
1cThe obtained time constants (, , and τ_*S*_0__) are shown in Table 1.

**Table 1:** Quantum Yields, Excited State Lifetimes, and Exciton Localization Time Constants for the Studied Systems

System	Φ (%)^a^	(fs)^b^	(fs)^b^	τ_*S*_0__ (fs)^b^	τ_loc_ (fs)^c^
monomer, CIS	17 ± 4	1599	444	2398	—
monomer, CISD	22 ± 4	1125	439	1759	—
ring, CIS	16 ± 4	5652	338	6141	48 [45]
ring, CISD	34 ± 5	704	299	1073	52 [51]

a (*N* is the number of trajectories which relaxed to the ground state within 10 ps).

b See eq 1.

c The first number is obtained using eq 4; the second number (in square brackets) using eq 6.

For the monomer, the CIS and CISD population dynamics are qualitatively similar, with CIS being somewhat slower (e.g.,  ps vs  ps, see Table 1) and showing a residual ππ* population of 0.11 at 10 ps. Remarkably, a much larger difference is observed when comparing CIS and CISD population dynamics of trisazobenzenophane (Figure 1, right). The internal conversion occurs much faster at the CISD level ( ps vs  ps). Also, qualitatively different results are obtained when comparing the monomer and the ring: The nonadiabatic relaxation is decelerated at the CIS level, whereas it is accelerated at the CISD level (compare the time constants in Table 1). At the CISD level, there are more states between the initially excited state and the ground state than at the CIS level (see Figure 1 and Table S1). The larger density of many-electron states results in (on average) smaller energy gaps between the states and more (near-)crossings. It can thus be anticipated that nonadiabatic transitions will lead to a faster relaxation at the CISD level.

Having observed these differences in population dynamics, we proceeded by analyzing the exciton localization dynamics. To do so, we compute the one-particle spinless transition density matrix (TDM) (between the ground state and the current excited state) in atomic orbital basis *P*_*μν*_^[AO]^ as described in our previous work^23^ and further contract it to fragments (individual azobenzene units) to obtain a 3 × 3 matrix *F*_*XY*_:
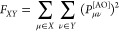
2The diagonal elements *F*_*XX*_ quantify contributions of local excitations (localized on fragment *X*), and off-diagonal elements *F*_*XY*_ (*Y* ≠ *X*) quantify charge-transfer excitations (between fragments *X* and *Y*). The CH_2_ linkers are excluded from the analysis.

Importantly, the TDM vanishes for purely doubly excited states.^29,30^ Therefore, we compute the sum *S* of matrix elements *F*_*XY*_ to assess the involvement of the doubly excited states in the nonadiabatic dynamics:
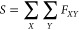
3

Since **P**^[AO]^ = **0** for a purely doubly excited state, we do not divide by  in eq 2 in contrast to our earlier definition of “fraction of transition density matrix” (FTDM).^23,24,31^

Further, to judge on exciton localization we analyze how the largest (or “highest”, H) diagonal element *F*_H_ changes with time.^8,32,33^ The ensemble-averaged *F*_H_ as a function of time is presented in Figure 3a. Importantly, the CIS and CISD *F*_H_(*t*) curves are similar. Both show the rise of *F*_H_ with a localization time constant (τ_loc_) of ∼50 fs (see Table 1) which was obtained using an exponential fit of the form
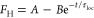
4applied to the first picosecond of the simulations. Thus, it appears that inclusion of double excitations does not considerably affect the time scale of the ultrafast exciton localization process.

**Figure 3:**
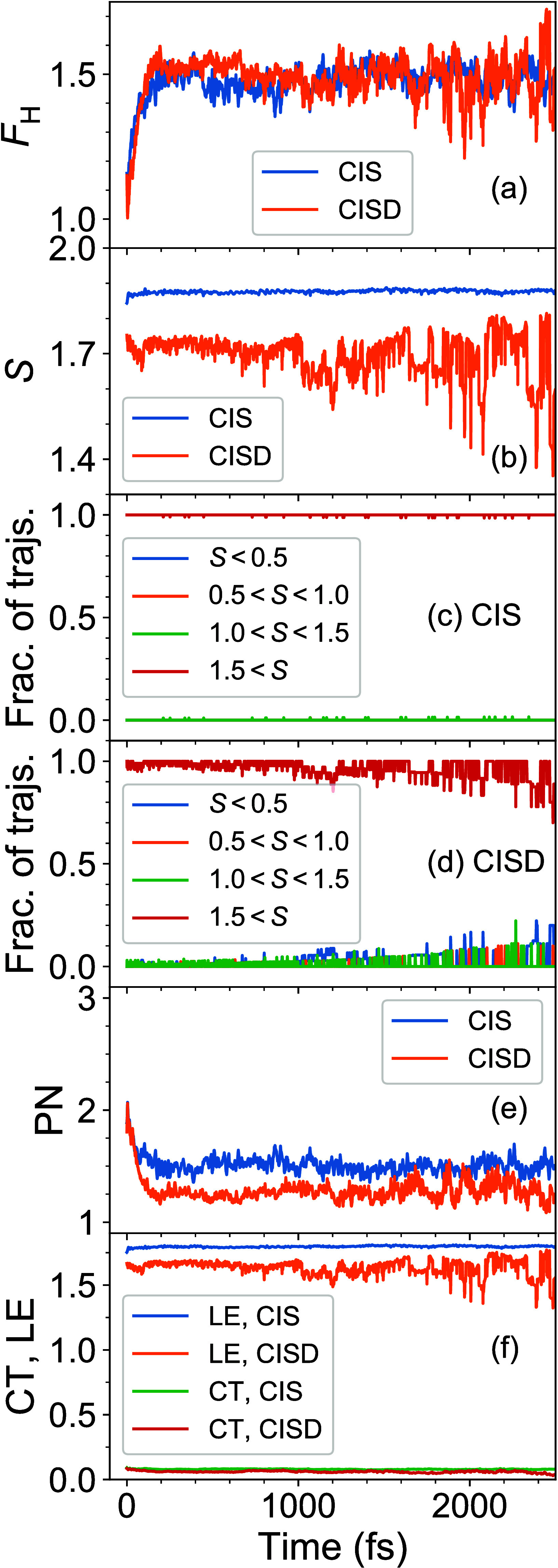
(a) Ensemble-averaged *F*_H_(*t*) for CIS and CISD. (b) Ensemble-averaged *S*(*t*) for CIS and CISD. (c, d) Fractions of trajectories with *S* values in certain intervals (as defined in the legends) for CIS (c) and CISD (d). (e) Ensemble-averaged PN(*t*) for CIS and CISD. (f) Ensemble-averaged LE(*t*) and CT(*t*) for CIS and CISD.

The ensemble-averaged *S* as a function of time is shown in Figure 3b. It is seen that (i) the CIS *S* values are larger than that of CISD and (ii) the CISD *S* curve is noisier. Fitting the first picosecond of the simulations with a constant, we obtain *S*^CIS^ ≈ 1.87 and *S*^CISD^ ≈ 1.72. To understand how the averaged curves are brought about, we calculated fractions of trajectories possessing an *S* value lying in a certain interval at a given time. Specifically, we count how many trajectories have (i) *S* < 0.5, (ii) 0.5 < *S* < 1.0, (iii) 1.0 < *S* < 1.5, and (iv) *S* > 1.5. The corresponding fractions are shown in Figure 3c,d for CIS and CISD, respectively. In both cases, the major fraction of trajectories corresponds to *S* > 1.5. Thus, on average, single excitations prevail during dynamics. At that, for CISD we also observe small but nonzero fractions with *S* < 1.

Exciton localization in multichromophoric systems is often quantified with a participation number (or delocalization length):^34^
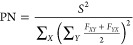
5

It is a scalar ranging from 1 (localization on one fragment) to 3 in our case (uniform delocalization over all three fragments). Equation 5 is, however, problematic in case *S* = 0 (or, equivalently, all *F*_*XY*_ = 0), i.e., for purely doubly excited states. But, as we have seen (Figure 3d), *S* = 0 is not the case for our system (for most of times, at least), and, in practice, even if *F*_*XY*_ are small they are nonzero. Thus, PN can be calculated, and it is shown in Figure 3e. Fitting the first picosecond of PN curves as
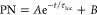
6we obtain τ_loc_ ≈ 45 fs and *B* ≈ 1.51 for CIS and τ_loc_ ≈ 51 fs and *B* ≈ 1.25 for CISD. Thus, this analysis also yields a similar localization time (∼50 fs) for CIS and CISD, with slightly larger extent of localization for CISD (corresponding to a smaller *B* value).

We also calculated measures of local excitations (LE) and charge transfer (CT) excitations as
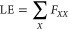
7a
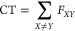
7bAs can be seen in Figure 3f, the CT contribution is minor at both the CIS and CISD levels of theory.

Further insight into the exciton dynamics can be obtained by inspection of the individual SH trajectories. In Figure 4, *F*_*XX*_(*t*) (*X* = 1, 2, 3), *S*(*t*), and the current state as a function of time are shown for selected single trajectories calculated with CIS (left column) and CISD (right column). From the *F*_*XX*_ curves it is seen that the exciton can flow from one monomer to another during dynamics in the ππ* manifold. Further, the *S* values are always large (almost 2) for the CIS trajectory, whereas “spikes” toward smaller values are observed in the *S* curve for the CISD trajectory. These spikes signify the participation of double excitations in the nonadiabatic dynamics. However, *S* remains large most of the time at the CISD level. This explains why the averaged *S* curve in Figure 3b passes at relatively high values (∼1.7).

**Figure 4:**
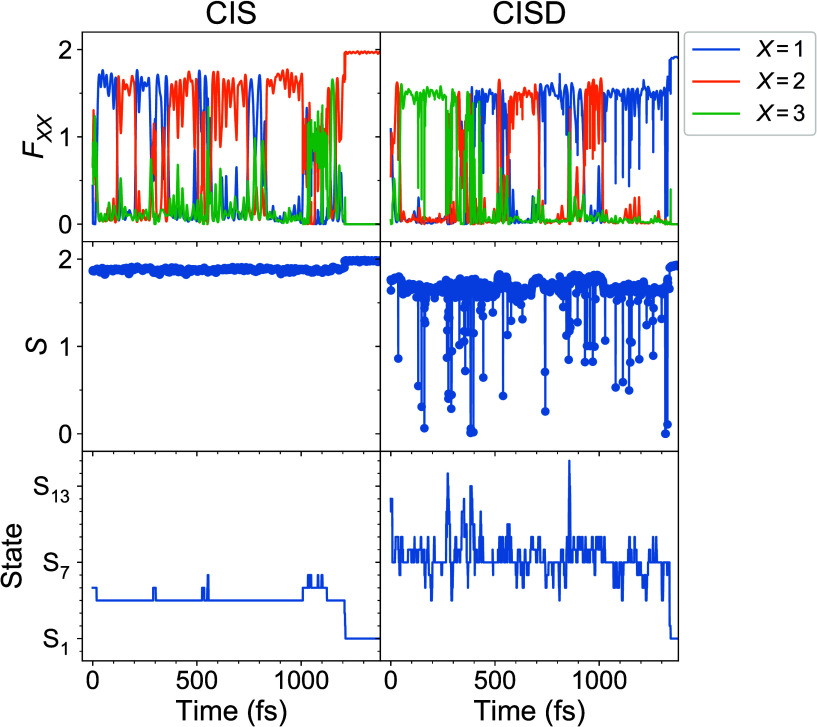
*F*_*XX*_(*t*) (*X* = 1, 2, 3) (top row), *S*(*t*) (middle row), and current state (*t*) (bottom row) for single trajectories calculated with CIS (left column) and CISD (right column).

Finally, we computed the quantum yields of the *trans* → *cis* isomerization. They are summarized in Table 1. At the CIS level, we find Φ ≈ 17% and Φ ≈ 16% for the monomer and the ring, respectively. At the CISD level, the quantum yields are higher: Φ ≈ 22% and Φ ≈ 34% for the monomer and the ring, respectively. In this respect, we note that inclusion of double excitations changes potential energy surfaces (PESs) to some extent (see Figure S11). Further, all reactive trajectories, i.e., those trajectories which show *trans* → *cis* switching, hop to the ground state at CNNC dihedral values <121° (see Figure S12). The unreactive trajectories, in turn, show hops either at CNNC values <121° or >121°, corresponding to “reactive” *trans* and “unreactive” *trans* pathways,^35,36^ respectively. For the monomer, we observe that more trajectories follow the “unreactive” *trans* pathway at the CIS level than at the CISD level (44% vs 33%, respectively). Moreover, at the CISD level, the quantum yield for trisazobenzenophane is higher than that for the monomer, whereas similar quantum yields are obtained for both systems at the CIS level. For the ring, we observe that more than 90% of trajectories follow the “reactive” pathway at both CIS and CISD levels, signifying the effect of the circular geometry. However, the branching to the *cis* and *trans* isomers is different (see Table S2).

Normally, if a trajectory is reactive, only one azobenzene unit undergoes isomerization (Figure S5). Some trajectories, however, exhibit upward hops from *S*_0_ (after initial internal conversion to *S*_0_) which may result in switching of the second unit (Figure S6). One trajectory with back hops (at the CISD level) demonstrates isomerization of all three azobenzene units (Figure S7). We should note that in our simulations the entire kinetic energy is available for compensation of potential energy variation upon hops, so only a few frustrated hops are observed in the case of the ring. Thus, the upward hops are expected to be exaggerated. Moreover, at the CISD level, five trajectories are trapped in a state with two CNNC angles oscillating near 90–100° (and the third one staying at ∼180°); see Figure S8.

Ensemble-averaged CNNC dihedral angle evolution curves for reactive and unreactive trajectories are shown in Figure S13. There it is seen that the *trans* → *cis* isomerization is (on average) the fastest for the ring at the CISD level, which correlates with the fastest internal conversion for this case (compare with Figure 2 and Table 1). We also note that, for the unreactive trajectories, the CNNC dihedral angles are smaller for the ring than for the monomer, which demonstrates the effect of the circular geometry.

In conclusion, we performed nonadiabatic surface hopping dynamics simulations for trisazobenzenophane (a molecular ring composed of three azobenzene units) at the rAM1/FOMO-CIS (referred to as “CIS” in this work) and rAM1/FOMO-CISD (“CISD”) levels of theory. The dynamical exciton localization was analyzed using a one-particle transition density matrix. Importantly, we found very similar exciton localization time scales with both CIS and CISD simulations, amounting to ∼50 fs for the studied multiazobenzene. In contrast, excited state lifetimes and isomerization quantum yields are affected much more strongly by inclusion of double excitations, with the former being shorter and the latter being higher at the CISD level in comparison to CIS.

## Methods

The electronic structures of studied azobenzenes were described with semiempirical configuration interaction (CI) [including either only single (S) or single and double (SD) excitations within an active orbital space] based on molecular orbitals (MO) obtained from a self-consistent field calculation with floating occupation (FO) numbers^37^ using Austin Model 1 (AM1)^38^ reparameterized (r) for azobenzene.^39^ The method is thus abbreviated as rAM1/FOMO-CI [more specifically, rAM1/FOMO-CIS and rAM1/FOMO-CISD for S and SD excitations, respectively]. The active space of six highest occupied (three π and three *n*) and three lowest unoccupied (three π*) orbitals was used for trisazobenzenophane. This results in 37 and 451 Slater determinants for CIS and CISD, respectively. For the monomer, the active space of two highest occupied (one π and one *n*) and one lowest unoccupied (one π*) orbitals was used [5 and 9 Slater determinants for CIS and CISD, respectively].

The nonadiabatic dynamics were modeled using the trajectory surface hopping (SH) approach^40^ combined with the semiempirical configuration interaction method,^41,42^ namely, rAM1/FOMO-CI introduced above. The so-called added potential^39^ (applied to each monomer) is used throughout but not the state-specific corrections.^39^ The initial conditions (geometries and velocities) were sampled from rAM1/FOMO-CI (CIS for subsequent CIS SH, and CISD for subsequent CISD SH) ground state Langevin trajectories propagated for 20 ps at *T* = 300 K with a time step of 0.1 fs. 100 geometries were selected starting at 2 ps and sampling every 180 fs. The SH trajectories were propagated for 10 ps with a time step of 0.1 fs. The energy-based decoherence correction was used to remedy overcoherence of the original surface hopping algorithm.^43^ The time-dependent electronic wave function was propagated using the local diabatization scheme.^41,44^ For trisazobenzenophane, 7 (*S*_0_–*S*_6_) and 16 (*S*_0_–*S*_15_) electronic states were included in the CIS and CISD simulations, respectively. For the monomer, three electronic states (*S*_0_–*S*_2_) were accounted for (for both the CIS and CISD). The nuclei were propagated classically on the on-the-fly calculated adiabatic rAM1/FOMO-CI PESs. The hopping probabilities were calculated using the prescription by Granucci and coauthors described in the appendix of ref (45). The calculations were done using a development version of MOPAC 2002^46^ including the surface hopping code of Granucci and Persico.^47^

## Supporting Information Available

Molecular orbitals, absorption spectra, initial populations, CNNC dihedral angles for selected trajectories, contributions of single and double excitations, potential energy curves, and analysis of structural dynamics (PDF)
Transparent Peer Review report available (PDF)
