# Supplementary material for: The Role of Double Excitations in Exciton Dynamics of Multiazobenzenes: Trisazobenzenophane as a Test Case
Source: J Phys Chem Lett. 2024 Jul 16;15(29):7482–8. doi: 10.1021/acs.jpclett.4c01608 (PMC11284852; doi:10.1021/acs.jpclett.4c01608)
Supplement: Supplementary file 1 — jz4c01608_si_001.pdf [file jz4c01608_si_001.pdf]

Supporting Information for "The Role of Double Excitations in Exciton Dynamics of Multiazobenzenes: Trisazobenzenophane as a Test Case"

Evgenii Titov*

University of Potsdam, Institute of Chemistry, Theoretical Chemistry, Karl-Liebknecht-Straße 24-25, 14476 Potsdam, Germany

E-mail: titov@uni-potsdam.de

Contents

S1 Molecular orbitals **S2**

S2 Absorption spectra **S3**

S3 Initial populations **S6**

S4 CNNC dihedral angles for selected trajectories **S7**

S5 Contributions of single and double excitations **S11**

S6 Potential energy curves **S14**

S7 Analysis of structural dynamics **S15**

S1 Molecular orbitals

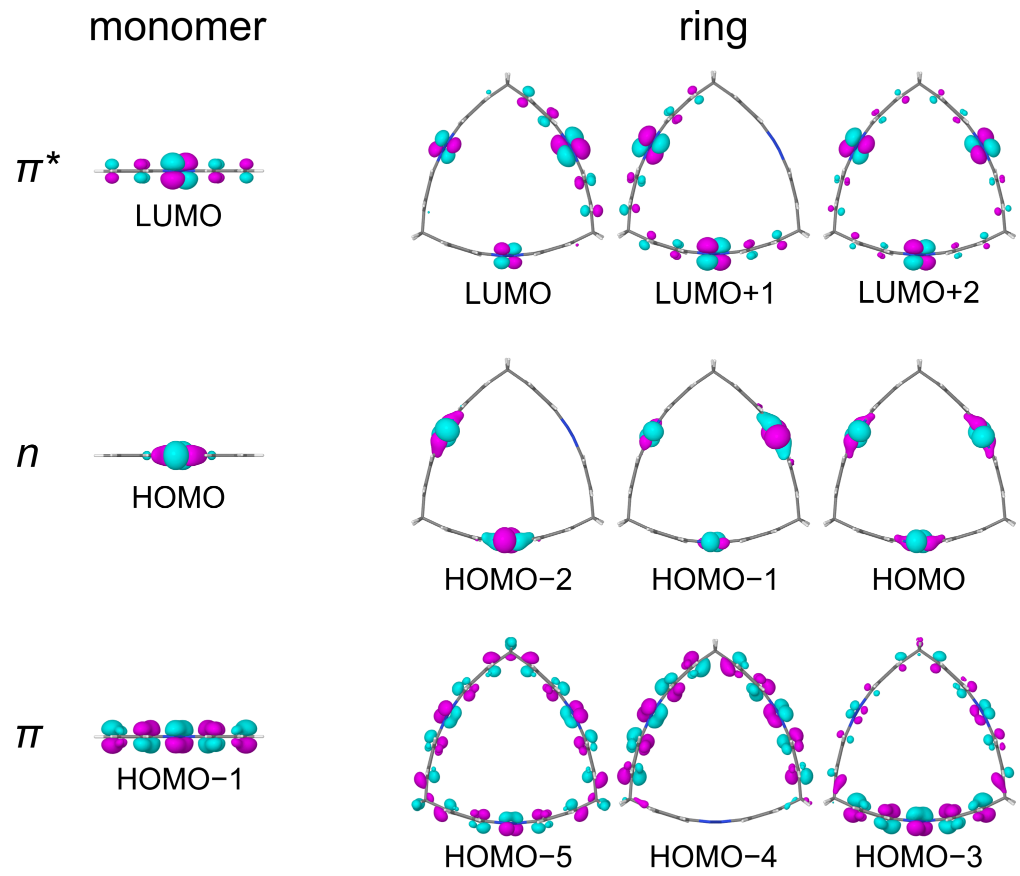

Figure S1: Molecular orbitals of the monomer (left) and the ring (right) used in the active space. The calculations are done at the CISD ground-state optimized geometries.

S2 Absorption spectra

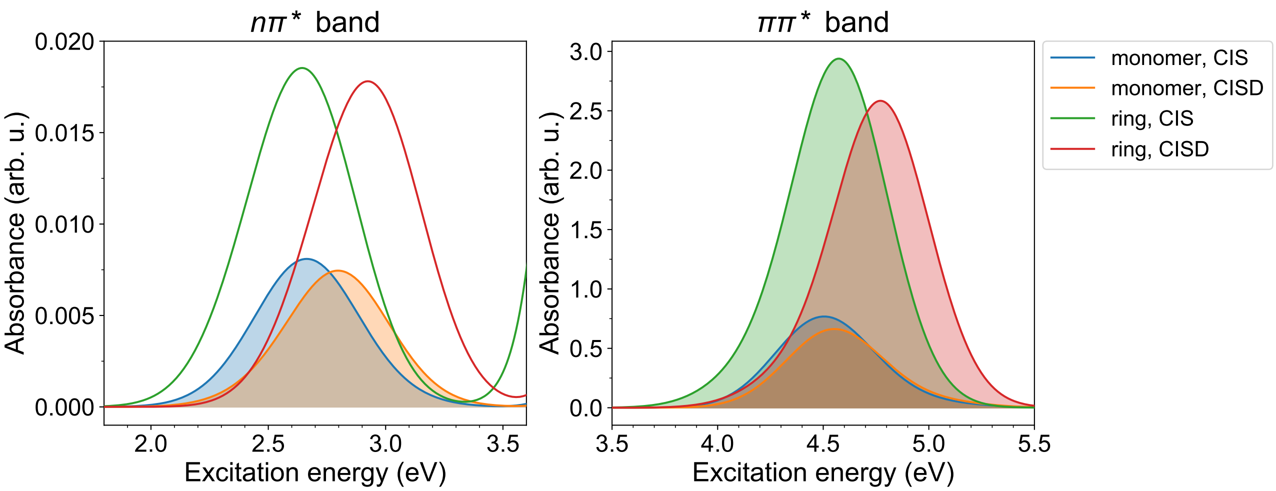

Figure S2: The $n\pi^*$ (left) and $\pi\pi^*$ (right) absorption bands of the studied systems. The spectra were calculated as $I(E) = \frac{1}{N_{\text{sn}}} \sum_{\alpha=1}^{N_{\text{sn}}} \sum_{i=1}^{N_{\text{st}}} f_{i,\alpha} \exp\left(-\frac{1}{2\gamma^2}\left(E - E_{i,\alpha}\right)^2\right)$. Here, I is absorbance, E is excitation energy, $N_{\text{sn}} = 100$ is the number of selected snapshots, N_{st} is the number of excited singlet states ($N_{\text{st}} = 2;\ 5;\ 18;$ and 20 for monomer, CIS; monomer, CISD; ring, CIS ; and ring, CISD, respectively), $E_{i,\alpha}$ and $f_{i,\alpha}$ are the excitation energy and oscillator strength, respectively, for the $S_0 \rightarrow S_i$ transition, for snapshot α, and $\gamma = 0.18598$ eV (1500 cm^{-1}) is a broadening parameter

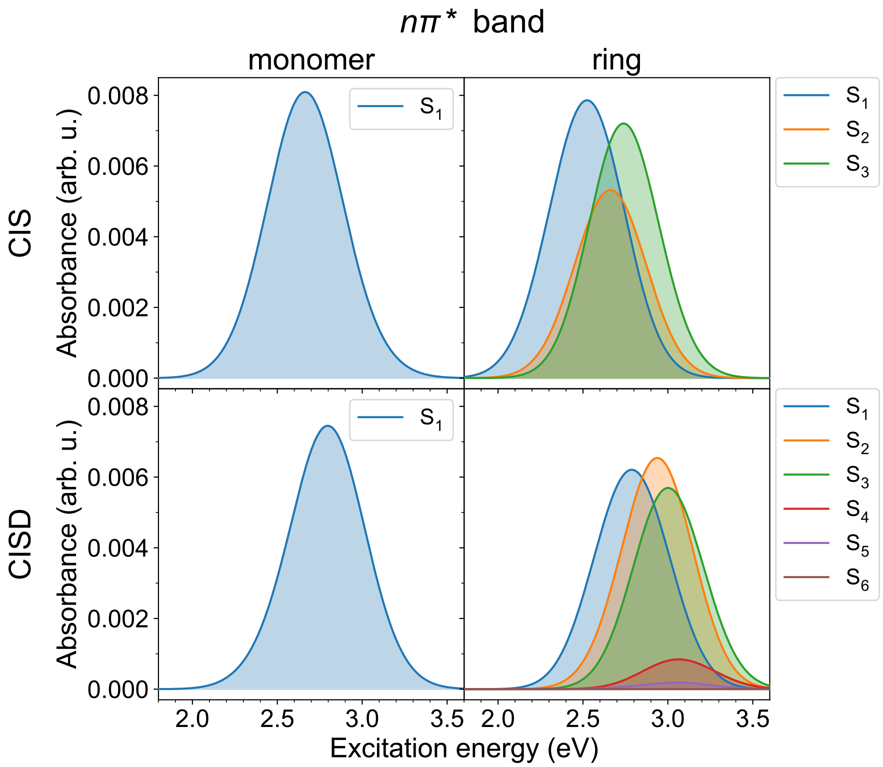

Figure S3: Contributions of individual states to the $n\pi^*$ band, calculated as $I_i(E) = \frac{1}{N_{\text{sn}}} \sum_{\alpha=1}^{N_{\text{sn}}} f_{i,\alpha} \exp\left(-\frac{1}{2\gamma^2}\left(E - E_{i,\alpha}\right)^2\right)$. See caption of Fig. S2 for further details.

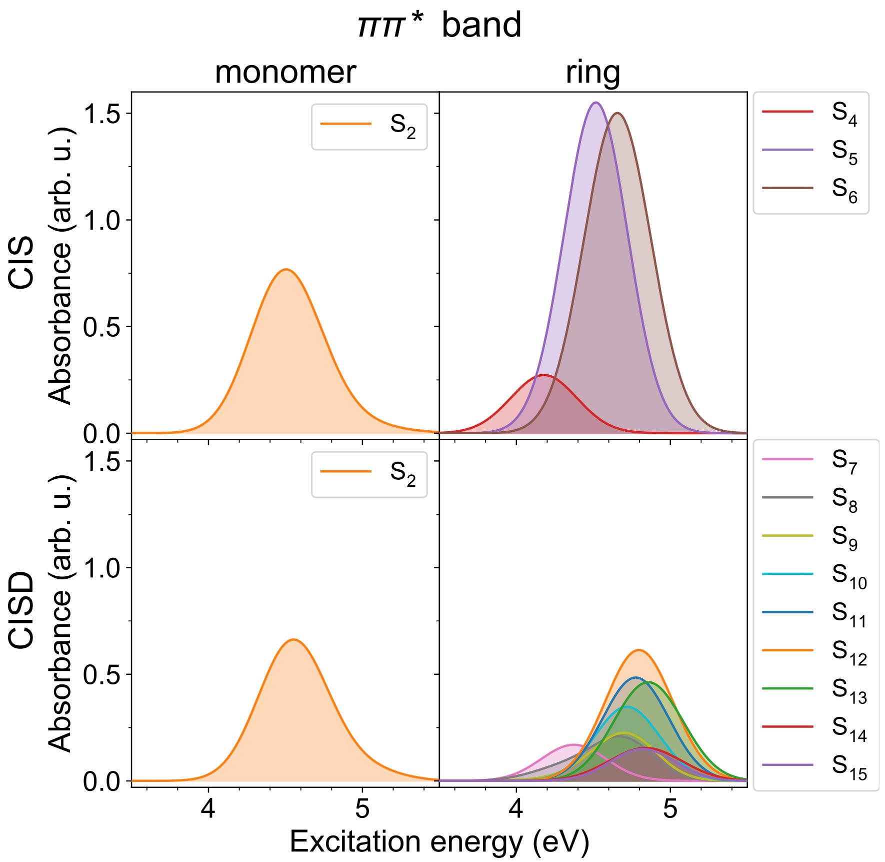

Figure S4: Contributions of individual states to the $\pi\pi^*$ band, calculated as $I_i(E) = \frac{1}{N_{\mathrm{sn}}} \sum_{\alpha=1}^{N_{\mathrm{sn}}} f_{i,\alpha} \exp\left(-\frac{1}{2\gamma^2}(E - E_{i,\alpha})^2\right)$. See caption of Fig. S2 for further details.

S3 Initial populations

Table S1: Initial populations (at $t = 0$) according to the sampling method of the brightest state.

	monomer		ring	
state	CIS	CISD	CIS	CISD
S_1				
S_2	1.00	1.00		
S_3				
S_4				
S_5			0.44	
S_6			0.56	
S_7				
S_8				0.06
S_9				0.08
S_{10}				0.12
S_{11}				0.16
S_{12}				0.24
S_{13}				0.16
S_{14}				0.11
S_{15}				0.07

S4 CNNC dihedral angles for selected trajectories

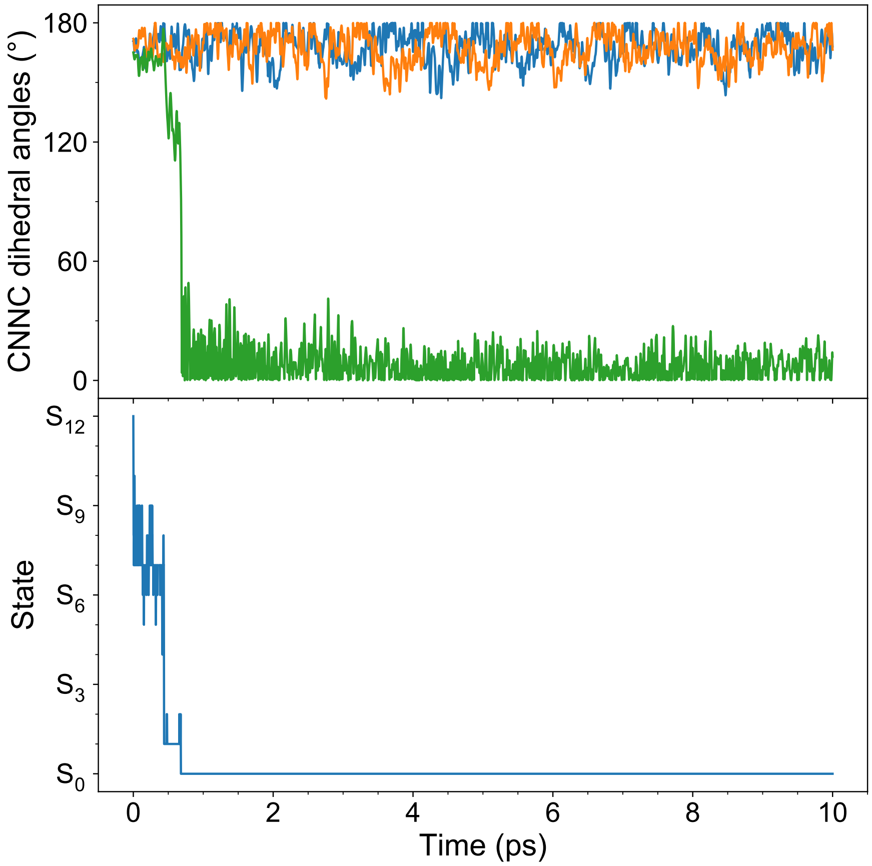

Figure S5: Example of a reactive CISD trajectory showing isomerization of one azobenzene unit. Top panel shows three CNNC dihedrals and the bottom panel the current state.

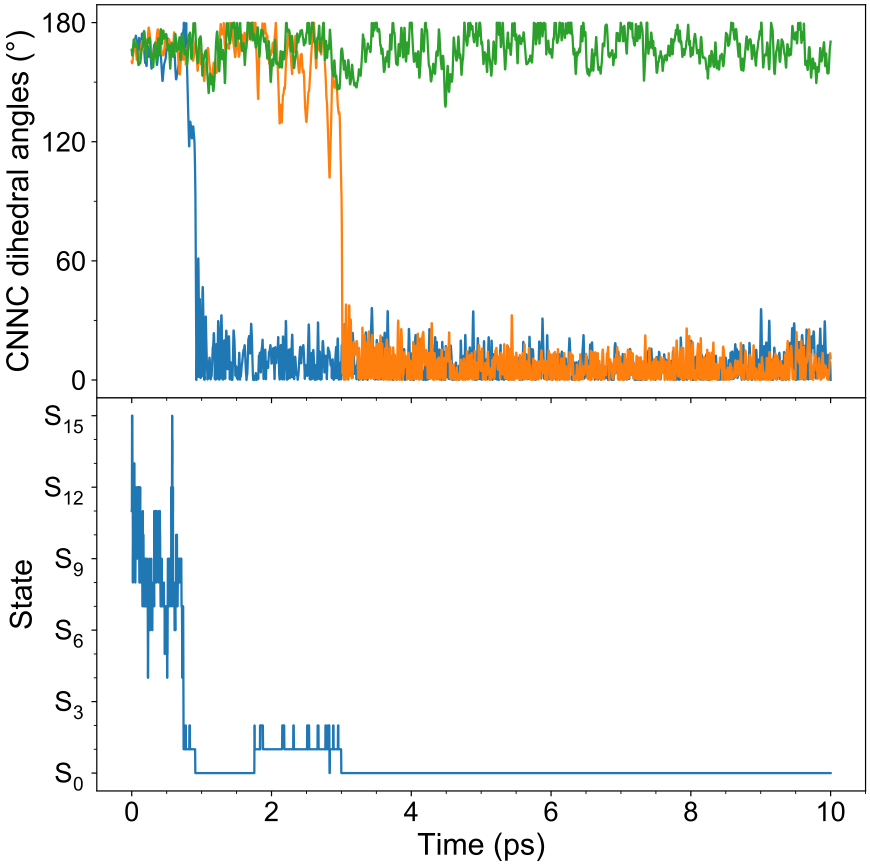

Figure S6: Example of a reactive CISD trajectory showing isomerization of two azobenzene units. Top panel shows three CNNC dihedrals and the bottom panel the current state.

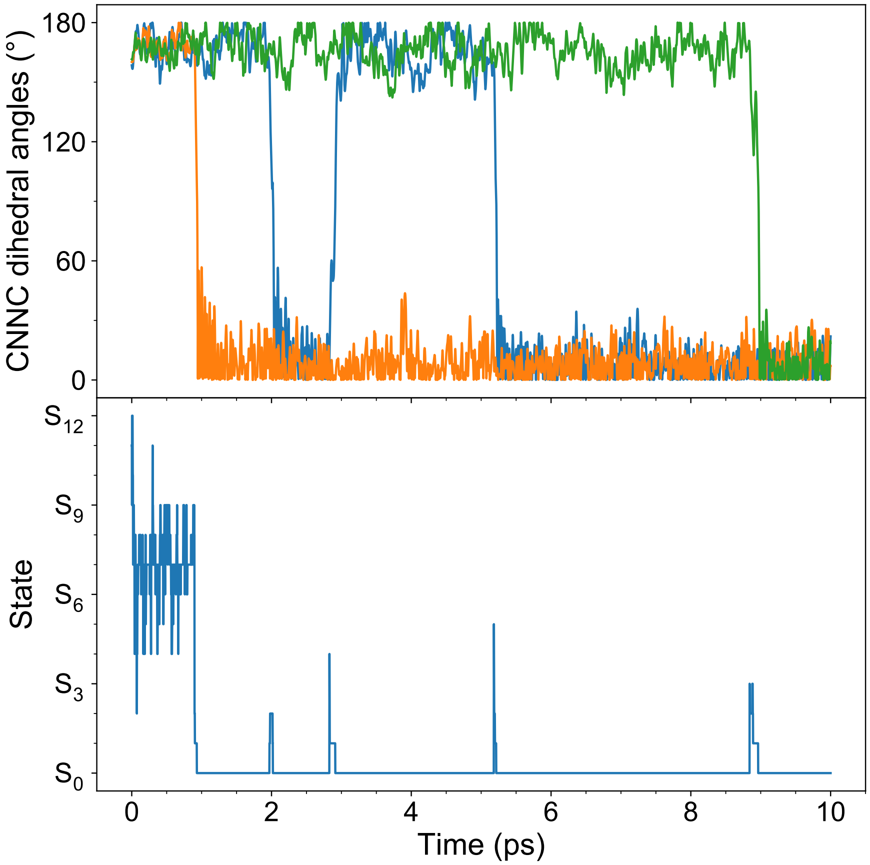

Figure S7: Reactive CISD trajectory showing isomerization of three azobenzene units. Top panel shows three CNNC dihedrals and the bottom panel the current state.

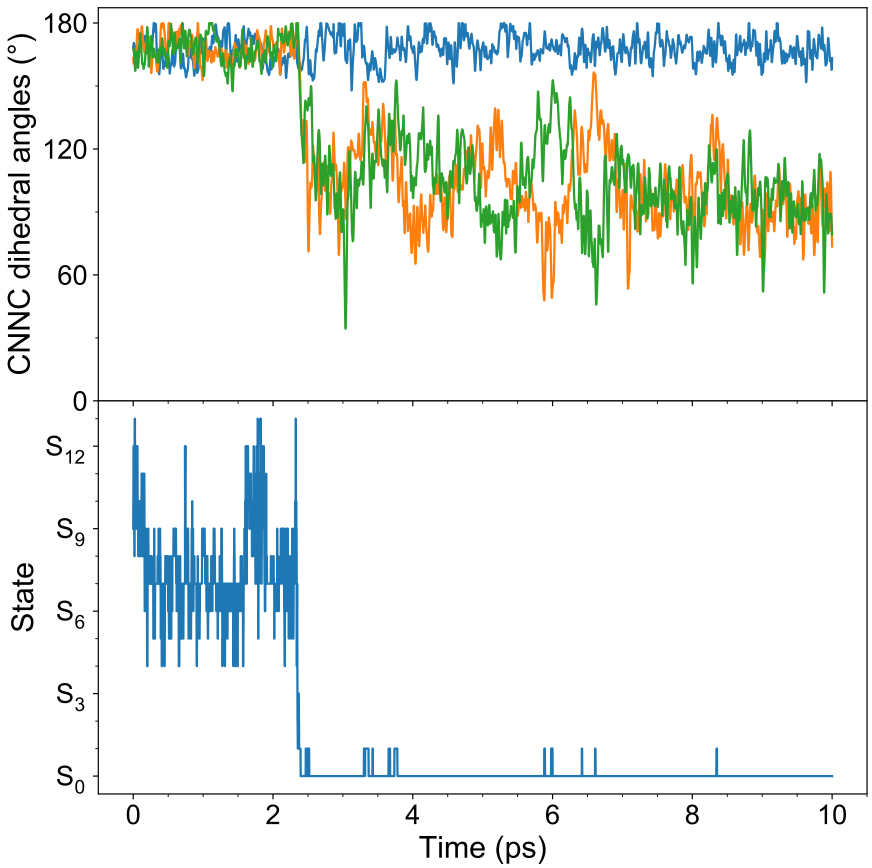

Figure S8: Example of a CISD trajectory showing two azobenzene units with CNNC dihedrals oscillating near 90–100°. Top panel shows three CNNC dihedrals and the bottom panel the current state.

S5 Contributions of single and double excitations

The current state wave function can be written as a sum of the singles (S) contribution (in which we also include the contribution of the reference, unexcited determinant) and the doubles (D) contribution:

$$\Phi = \sum_i C_i^{(\mathrm{S})} \Phi_i^{(\mathrm{S})} + \sum_j C_j^{(\mathrm{D})} \Phi_j^{(\mathrm{D})} \tag{S1}$$

The overall contribution of singles can then be quantified as $\sum_i \left| C_i^{(\mathrm{S})} \right|^2$. This quantity averaged over a swarm of trajectories (for the ring at the CISD level) is plotted in Fig. S9, top.

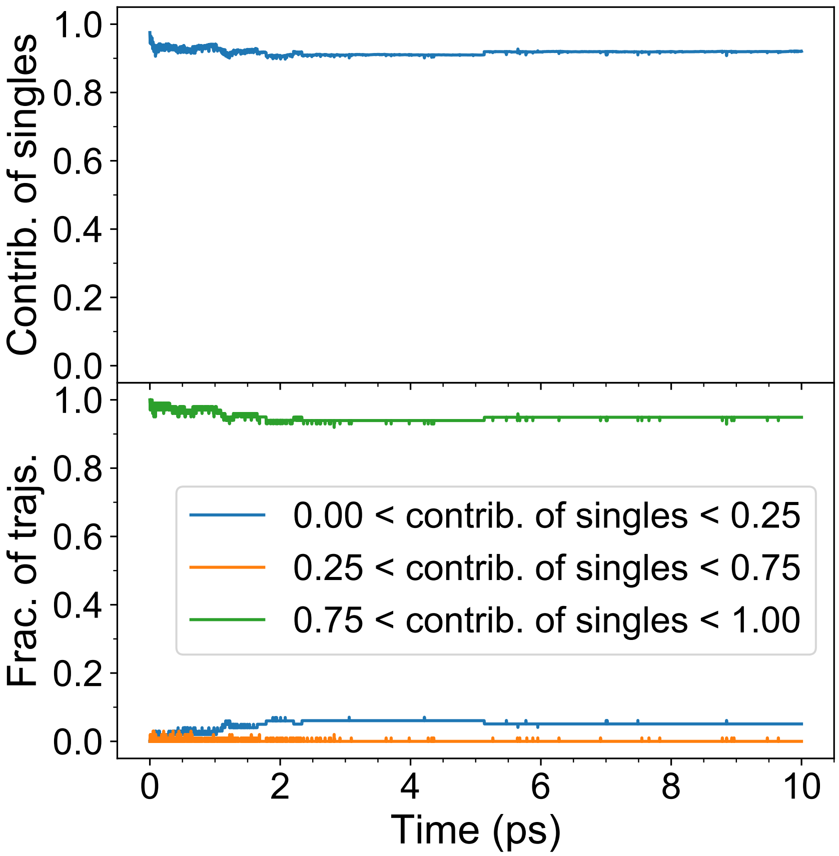

Figure S9: Ensemble-averaged contribution of singles (top panel) and fractions of trajectories with the contribution of singles in certain intervals (as defined in the legend) (bottom panel) for the ring at the CISD level.

It is seen that the contribution of singles is on average > 0.9. In addition, in Fig. S9, bottom, we plot fractions of trajectories with a certain contribution of singles, namely (i) smaller than 0.25, (ii) between 0.25 and 0.75, and (iii) larger than 0.75. Fraction (iii) is larger than 0.9 at all times, fraction (ii) reaches a maximal value of 0.03 at short times, and fraction (i) is 0.05 at the end of the simulation. Thus, single excitations clearly dominate the dynamics. Interestingly, fraction (i) at long times stems from trajectories with CNNC dihedrals oscillating near 90–100° (see Fig. S8). The corresponding doubly excited states are expected to be the singlet correlated triplet pair (TT) states.[S1]

Further, one can perform more detailed analysis rewriting the wave function as:

$$\Phi = C_0\Phi_0 + \sum_i C_i^{(S1)}\Phi_i^{(S1)} + \sum_j C_j^{(S2)}\Phi_j^{(S2)} + \sum_k C_k^{(D1)}\Phi_k^{(D1)} + \sum_l C_l^{(D2)}\Phi_l^{(D2)} \tag{S2}$$

Here, we distinguish between the reference contribution ($C_0\Phi_0$), "singles 1" (S1) contribution including single excitations from (HOMO−2, HOMO−1, HOMO) [the n orbitals at the ground-state minimum geometry] to (LUMO, LUMO+1, LUMO+2), "singles 2" (S2) contribution including single excitations from (HOMO−5, HOMO−4, HOMO−3) [the π orbitals at the ground-state minimum geometry] to (LUMO, LUMO+1, LUMO+2), "doubles 1" (D1) contribution including double excitations from (HOMO−2, HOMO−1, HOMO) to (LUMO, LUMO+1, LUMO+2) [*i.e.*, $(nn)(\pi^*\pi^*)$ excitations at the ground-state minimum geometry], and "doubles 2" (D2) contribution including double excitations from (HOMO−5, HOMO−4, HOMO−3) to (LUMO, LUMO+1, LUMO+2) [*i.e.*, $(n\pi)(\pi^*\pi^*)$ and $(\pi\pi)(\pi^*\pi^*)$ excitations at the ground-state minimum geometry]. Each contribution can be quantified by the sum of the corresponding squared coefficients, and the contributions obtained in this way and averaged over a swarm of trajectories are shown in Fig. S10. It is seen again that doubles provide a minor contribution. Interestingly, for the ring, we see that singles 1 contribution is on average larger than singles 2 contribution at longer times (particularly, for CIS). In this regard, we note that orbital character changes with geometry, *e.g.*, HOMO may acquire a

substantial π character. This should be kept in mind when comparing to Fig. 2 of the main text (*e.g.*, there a $\pi\pi^*$ population of 0.2 is observed for the ring at the CIS level at 10 ps).

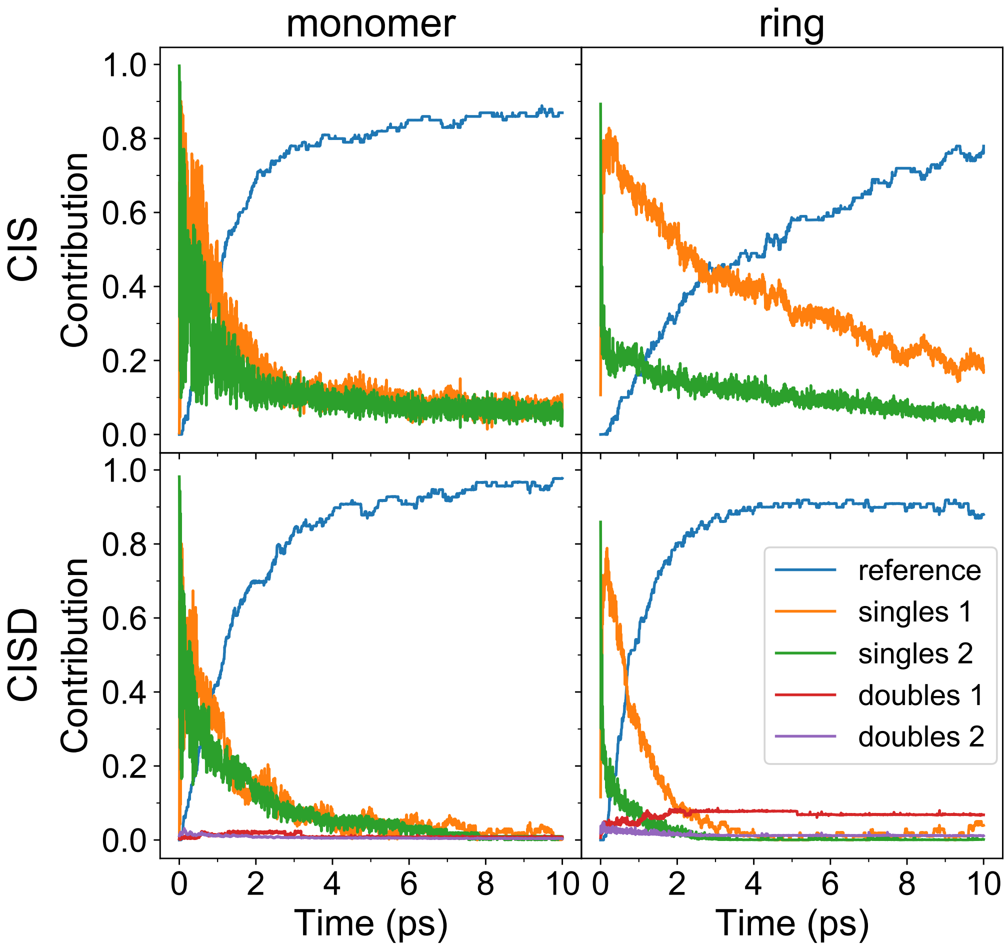

Figure S10: Ensemble-averaged contributions of the reference, singles 1, singles 2, doubles 1, and doubles 2 groups. See text for the details.

S6 Potential energy curves

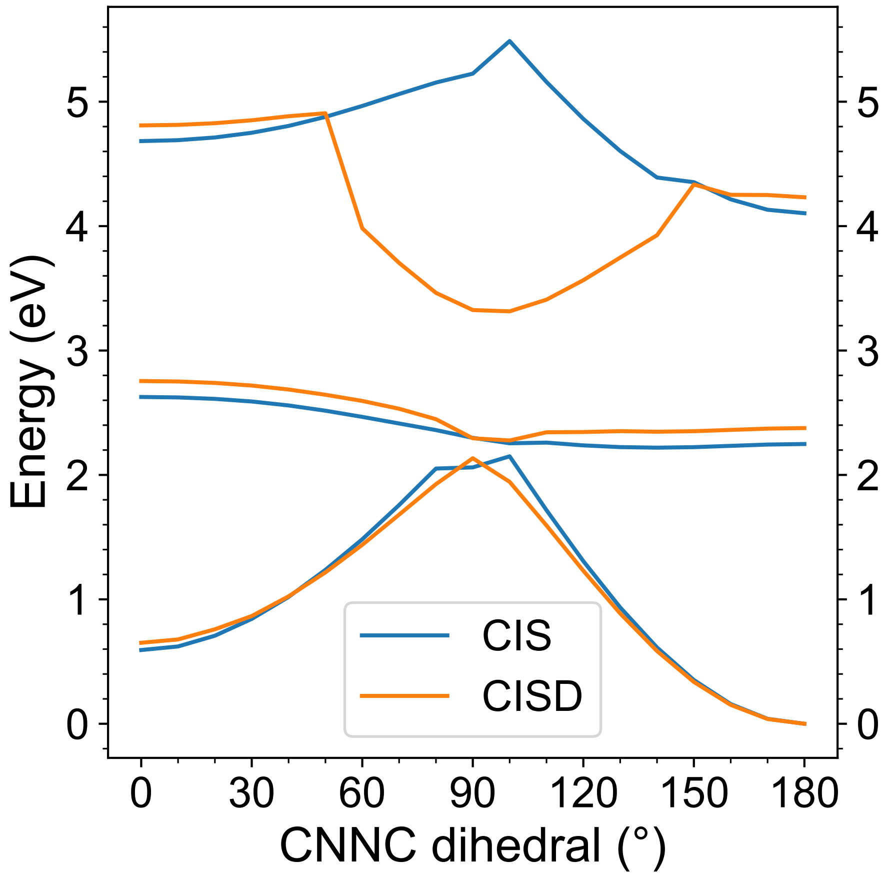

Figure S11: Potential energy curves (relaxed scans with a step of 10°) along the CNNC dihedral angle for the monomer at the CIS and CISD levels. The dip in the S_2 curve around 90° stems from double excitations.

S7 Analysis of structural dynamics

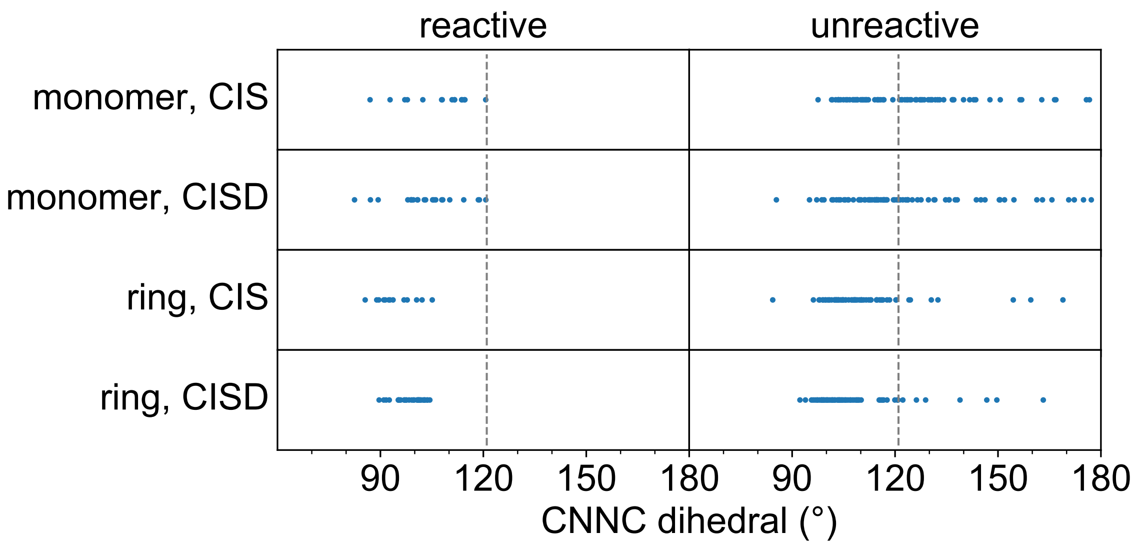

Figure S12: CNNC dihedral angles just after a hop to the ground state for the reactive (left) and unreactive (right) trajectories. For the ring, in the case of unreactive trajectories, the minimal of the three CNNC dihedrals was used. The vertical dashed lines show the CNNC value of 121°.

Table S2: Fractions of trajectories (in % with respect to the total number of trajectories which reached the ground state) corresponding to three decay pathways: (i) *cis* — reactive pathway leading to the *cis* isomer, (ii) "reactive" *trans* — unreactive pathway through the "reactive region" (CNNC dihedral $< 121°$) leading to the *trans* isomer, and (iii) "unreactive" *trans* — unreactive pathway through the "unreactive region" (CNNC dihedral $> 121°$) leading to the *trans* isomer.

System	*cis*	"reactive" *trans*	"unreactive" *trans*
monomer, CIS	17	39	44
monomer, CISD	22	44	33
ring, CIS	16	76	9
ring, CISD	34	59	7

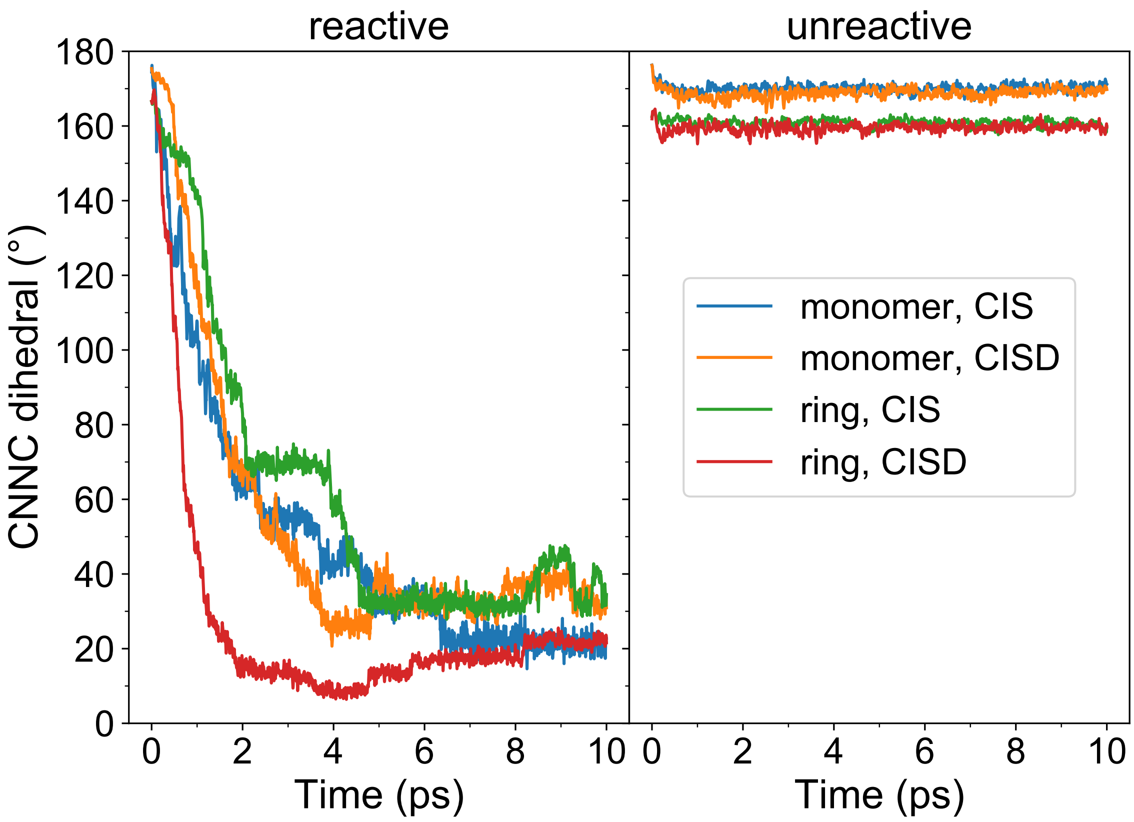

Figure S13: Ensemble-averaged CNNC dihedral angles as a function of time for the reactive (left) and unreactive (right) trajectories. For the ring, in the case of unreactive trajectories, the minimal of the three CNNC dihedrals was used.

References

(S1) Gil, E. S.; Granucci, G.; Persico, M. Surface Hopping Dynamics with the Frenkel Exciton Model in a Semiempirical Framework. *J. Chem. Theory Comput.* **2021**, *17*, 7373–7383.
