# Supplementary material for: The Role of Double Excitations in Exciton Dynamics of Multiazobenzenes: Trisazobenzenophane as a Test Case
Source: J Phys Chem Lett. 2024 Jul 16;15(29):7482–8. doi: 10.1021/acs.jpclett.4c01608 (PMC11284852; doi:10.1021/acs.jpclett.4c01608)
Supplement: Supplementary file 2 — jz4c01608_si_002.pdf [file jz4c01608_si_002.pdf]

jz-2024-016084.R1

Name: Peer Review Information for "The Role of Double Excitations in Exciton Dynamics of Multiazobenzenes: Trisazobenzenophane as a Test Case"

First Round of Reviewer Comments

Reviewer: 1

Comments to the Author

The manuscript is devoted to computational investigation of the excited state dynamics of a multichromophoric system consisting of three azobenzene subunits. In particular, the aim is to show how the inclusion of double excitations in a CIS approach affects the dynamics of the system. The manuscript is well written and deserves to be published. Here are my remarks.

1) The author shows that the inclusion of double excitations gives rise to a faster dynamics (especially for the trimer). However, the reason for this behavior is not discussed. The author should try to provide an explanation for this effect.

2) The inclusion of double excitations leads to an increase of the trans->cis photoisomerization quantum yield. However, usually in azobenzene a faster decay corresponds to a decrease in the photoisomerization quantum yield.
So, what is the origin of the increase in the quantum yield?

3) Some trajectories give rise to backward hops from S0 to the upper states. Given the extent of the system, if all the kinetic energy is available for backward jumps, there will not be any forbidden hop. This matter should be briefly discussed by the author.

Reviewer: 2

Comments to the Author

In this work, semiempirical calculations on the photo dynamics of trisazobenzenophane, a ring system containing three azobenzene units, are reported. The analysis of the photo dynamics is performed by means of several descriptors which should show (i) the contributions of double excitations used for the description of the wave function in comparison to including only single excitations and (ii) to describe the dynamics itself. In summary, the work performed is interesting even though very technical. I have a few comments which the author should consider before acceptance of the paper.

Comments:

1. The comparison of CIS vs. CISD results on the photo dynamics plays a major role in this paper. Unfortunately, all configurations e.g. of pi-pi* (single excitations) and (n-pi)(pi*-pi*) (double excitations) are arranged in one group (Fig. 1). The importance of the double excitations is evaluated a bit indirectly by the descriptor S. I wanted to ask the author to generate separate groups for the different types of excitations and show the electronic state populations for the extended list of groups beyond what is shown in Fig. 2. In this way one should be able to analyze the importance of the double excitations directly.

2. As already mentioned, the paper shows a number of descriptors to describe the localization of the excitations and related properties. A comparison of the trimer with the monomer is given always. What I am missing is a detailed structural comparison of the monomer dynamics and that in the trimer. How does the ring formation affect the flexibility of the deactivation to the ground state? How are the quantum yields affected?

3. Figure S4 and Table S1 show the contributions of the individual states to the spectra and as a result the initial populations for the dynamics. Table S1 shows for CISD a distribution over states S8 to S15. In Fig. 1 the oscillator strengths (CISD) are given where for states S8 to S15 the two highest pi-pi* states dominate by far and the double excitations are dark. This seems to me to be a significant contradiction to the almost equal distribution of the initial states for the dynamics. According to Fig. 1, the dynamics should start predominantly from states S14 and S15 (I assume these are the numbers of the two bright states). However, they rank among the weakest ones. I think this is an important point and contradiction, which should be clarified since it could affect the entire photo dynamics based on the CISD wave function.

Author's Response to Peer Review Comments:

Dear Editor,

Thank you for handling this submission.

We have addressed the requested scientific and non-scientific changes.

The reply to the reviewers' comments is attached.

We look forward to hearing from you soon.

Sincerely,

Dr. Evgenii Titov

Reply to the reviewers, changes made to the manuscript

We thank the reviewers for the careful reading of the manuscript, and for the useful comments. We have tried to address every point raised by the reviewers, and made corresponding changes to improve the manuscript. In the following, the points raised by the referees and our responses to them are listed in detail.

Reviewer 1

The manuscript is devoted to computational investigation of the excited state dynamics of a multichromophoric system consisting of three azobenzene subunits. In particular, the aim is to show how the inclusion of double excitations in a CIS approach affects the dynamics of the system. The manuscript is well written and deserves to be published.

Author reply:
We thank the reviewer for the positive evaluation of the manuscript and the provided comments/questions.

Here are my remarks:

1. *The author shows that the inclusion of double excitations gives rise to a faster dynamics (especially for the trimer). However, the reason for this behavior is not discussed. The author should try to provide an explanation for this effect.*

   Author reply:

   At the CISD level, there are more states between the initially excited state and the ground state than at the CIS level (see Fig. 1 and Tab. S1). The larger density of many-electron states results in (on average) smaller energy gaps between the states and more (near-)crossings. It can thus be anticipated that nonadiabatic transitions will lead to a faster relaxation at the CISD level.

   Changes made to the manuscript:

We have added the following explanation on Page 7:

"At the CISD level, there are more states between the initially excited state and the ground state than at the CIS level (see Fig. 1 and Tab.

S1). The larger density of many-electron states results in (on average) smaller energy gaps between the states and more (near-)crossings. It can thus be anticipated that nonadiabatic transitions will lead to a faster relaxation at the CISD level."

2. *The inclusion of double excitations leads to an increase of the trans→cis photoisomerization quantum yield. However, usually in azobenzene a faster decay corresponds to a decrease in the photoisomerization quantum yield. So, what is the origin of the increase in the quantum yield?* **Author reply:**

The inclusion of double excitations modifies, in general, potential energy surfaces. We have calculated relaxed scans along the CNNC dihedral angle to demonstrate this point (new Fig. S11). Moreover, we have analyzed hopping geometries for hops to the ground state. All reactive trajectories hop to the ground state at CNNC dihedral values < 121 $^\circ$. The unreactive trajectories, in turn, show hops either at CNNC values < 121 $^\circ$ or > 121 $^\circ$, corresponding to "reactive" *trans* and "unreactive" *trans* pathways, respectively. For the monomer, more trajectories follow the "unreactive" *trans* pathway at the CIS level than at the CISD level. For the ring, the majority of trajectories follows the "reactive" pathway at both CIS and CISD level, signifying the effect of the circular geometry. However, the branching to the *cis* and *trans* isomers is different when comparing CIS to CISD. The quantitative analysis is provided in new Fig. S12 and Tab. S2.

Changes made to the manuscript:

We have added new Figs. S11 (potential energy curves) and S12 (CNNC values for hopping geometries) and new Tab. S2 (analysis of decay pathways) to the SI. We have added the following on Pages 12–13 of the main text:

"In this respect, we note that inclusion of double excitations changes potential energy surfaces (PESs) to some extent (see Fig S11). Further, all reactive trajectories, *i.e.*, those trajectories which show *trans* → *cis* switching, hop to the ground state at CNNC dihedral values < 121 $^\circ$ (see Fig. S12). The unreactive trajectories, in turn, show hops either at CNNC values < 121 $^\circ$ or > 121 $^\circ$, corresponding to "reactive" *trans* and "unreactive" *trans* pathways,[35,36] respectively. For the monomer, we observe that more trajectories follow the "unreactive" *trans* pathway at the CIS level than at the CISD level (44% vs. 33%, respectively)." and

"For the ring, we observe that more than 90% of trajectories follow the "reactive" pathway at both CIS and CISD levels, signifying the effect of the circular geometry. However, the branching to the *cis* and *trans* isomers is different (see Tab. S2)."

3. *Some trajectories give rise to backward hops from S0 to the upper states. Given the extent of the system, if all the kinetic energy is available for backward jumps, there will not be any forbidden hop. This matter should be briefly discussed by the author.*

Author reply:

We agree. In our simulations the entire kinetic energy is available to correct for the change in the potential energy. In fact, there are only 3 frustrated hops for the azobenzene ring (trimer) [1 at the CIS level and 2 at the CISD level] in contrast to 26 for the monomer [10 at the CIS level and 16 at the CISD level].

Changes made to the manuscript:

We have added the following on Page 13:

"We should note that in our simulations the entire kinetic energy is available for compensation of potential energy variation upon hops, so only few frustrated hops are observed in the case of the ring. Thus, the upward hops are expected to be exaggerated."

Reviewer 2

In this work, semiempirical calculations on the photo dynamics of trisazobenzenophane, a ring system containing three azobenzene units, are reported. The analysis of the photo dynamics is performed by means of several descriptors which should show (i) the contributions of double excitations used for the description of the wave function in comparison to including only single excitations and (ii) to describe the dynamics itself. In summary, the work performed is interesting even though very technical. I have a few comments which the author should consider before acceptance of the paper.

Author reply:

We thank the reviewer for the positive evaluation of the manuscript and the provided comments/questions.

Comments:

1. *The comparison of CIS vs. CISD results on the photo dynamics plays a major role in this paper. Unfortunately, all configurations e.g. of pi-pi* (single excitations) and (n-pi)(pi*-pi*) (double excitations) are arranged in one group (Fig. 1). The importance of the double excitations is evaluated a bit indirectly by the descriptor S. I wanted to ask the author to generate separate groups for the different types of excitations and show the electronic state populations for the extended list of groups beyond what is shown in Fig. 2. In this way one should be able to analyze the importance of the double excitations directly.*

   Author reply:

   We have analyzed the contributions of single and double excitations as presented in new sec. S5 of the SI. Specifically, we partitioned the current state electronic wave function in terms of singles and doubles, and evaluated their individual contributions during dynamics (considering a swarm of trajectories). This analysis demonstrates the dominance of the single excitations throught the dynamics as well as reveals quantitatively the contribution of doubles. Please see sec. S5 for details.

   Changes made to the manuscript:

We have added new sec. S5 (with new Figs. S9 and S10) to the SI and the following on Page 5 of the main text:

"Detailed analysis of contributions of single and double excitations is provided in section S5 of SI. This analysis shows the dominance of the single excitations throughout the dynamics (see Figs. S9 and S10)."

2. *As already mentioned, the paper shows a number of descriptors to describe the localization of the excitations and related properties. A comparison of the trimer with the monomer is given always. What I am missing is a detailed structural comparison of the monomer dynamics and that in the trimer. How does the ring formation affect the flexibility of the deactivation to the ground state? How are the quantum yields affected?*

Author reply:

We have added new section S7 to the SI which summarizes analysis of structural dynamics. In particular, we have analyzed the hopping geometries for hops to the ground state as well as the evolution of ensemble-averaged CNNC dihedral angles — the primary coordinate for the isomerization. As described above (in the reply to point 2 of Reviewer 1), all reactive trajectories hop to the ground state at CNNC dihedral values $< 121^\circ$. The unreactive trajectories, in turn, show hops either at CNNC values $< 121^\circ$ or $> 121^\circ$, corresponding to "reactive" *trans* and "unreactive" *trans* pathways, respectively. For the monomer, more trajectories follow the "unreactive" *trans* pathway at the CIS level than at the CISD level. For the ring, the majority of trajectories follows the "reactive" pathway at both CIS and CISD level, signifying the effect of the circular geometry. However, the branching to the *cis* and *trans* isomers is different when comparing CIS to CISD.

Moreover, we have included ensemble-averaged CNNC evolution curves for the reactive and unreactive trajectories demonstrating (i) the fastest switching for the ring at the CISD level and (ii) in the case of the unreactive trajectories, smaller CNNC angles for the ring than for the monomer which shows the effect of ring formation. The quantum yields are summarized in Tab. 1.

Changes made to the manuscript:

We have added new Figs. S12 (CNNC values for hopping geometries) and S13 (ensemble-averaged CNNC evolution curves) and new Tab. S2 (analysis of decay pathways) to the SI. We have added the following on Pages 12–13 of the main text:

"Further, all reactive trajectories, *i.e.*, those trajectories which show *trans* → *cis* switching, hop to the ground state at CNNC dihedral values $< 121^\circ$ (see Fig. S12). The unreactive trajectories, in turn, show hops either at CNNC values $< 121^\circ$ or $> 121^\circ$, corresponding to "reactive" *trans* and "unreactive" *trans* pathways,[35,36] respectively. For the monomer, we observe that more trajectories follow the "unreactive" *trans* pathway at the CIS level than at the CISD level (44% vs. 33%, respectively)." and

"For the ring, we observe that more than 90% of trajectories follow the "reactive" pathway at both CIS and CISD levels, signifying the effect of the circular geometry. However, the branching to the *cis* and *trans* isomers is different (see Tab. S2)." and

"Ensemble-averaged CNNC dihedral angle evolution curves for reactive and unreactive trajectories are shown in Fig. S13. There it is seen that the *trans* → *cis* isomerization is (on average) the fastest for the ring at the CISD level, which correlates with the fastest internal conversion for this case (compare with Fig. 2 and Tab. 1). We also note that, for the unreactive trajectories, the CNNC dihedral angles are smaller for the ring than for the monomer which demonstrates the effect of the circular geometry."

3. *Figure S4 and Table S1 show the contributions of the individual states to the spectra and as a result the initial populations for the dynamics. Table S1 shows for CISD a distribution over states S8 to S15. In Fig. 1 the oscillator strengths (CISD) are given where for states S8 to S15 the two highest pi-pi* states dominate by far and the double excitations are dark. This seems to me to be a significant contradiction to the almost equal distribution of the initial states for the dynamics. According to Fig. 1, the dynamics should start predominantly from states S14 and S15 (I assume these are the numbers of the two bright states). However, they rank among the weakest ones. I think this is an important point and contradiction, which should be clarified since it could affect the entire photo dynamics based on the CISD wave function.*

Author reply:

Fig. S4 and Tab. S1 correspond to snapshots selected from groundstate Langevin trajectories (used to prepare initial conditions), whereas Fig. 1 is for the ground-state minimum geometry. The ordering of the excited states is very sensitive to geometry. It is, in fact, not surprising given the small energy gap between the $(n\pi)(\pi^*\pi^*)$ and $\pi\pi^*$ states observed in Fig. 1. Furthermore, the sampling method of the brightest state was used to select the initial electronic state. Which state is the brightest one (*i.e.*, the state corresponding to the largest oscillator strength) also depends very much on the geometry as reflected in Tab.
S1. In summary, there is no contradiction.

Changes made to the manuscript:

We have added the following on Page 5:

"We note that the ordering of the excited states is very sensitive to geometry. As a result, states S_8–S_{15} are initially populated at the
CISD level (Tab. S1)."
